# Postherpetic Abdominal Pseudohernia Evaluated by Standing Ultrasonography: A Case Report

**DOI:** 10.7759/cureus.110500

**Published:** 2026-06-08

**Authors:** Kosuke Sasaki, Tsunetaka Kijima, Yoko Fukuda, Kota Sakaguchi, Yoshihiko Shiraishi

**Affiliations:** 1 Internal Medicine, Okidozen Hospital, Oki-Dozen, JPN; 2 General Medicine, Shimane University Hospital, Izumo, JPN; 3 General Medicine, Kijima Clinic, Oda, JPN; 4 General Medicine, Okidozen Hospital, Oki-Dozen, JPN

**Keywords:** abdominal pseudohernia, abdominal wall bulging, point-of-care ultrasonography, segmental paralysis associated with herpes zoster, herpes zoster

## Abstract

Abdominal pseudohernia is a condition characterized by abdominal wall bulging without structural defects. We report a case of pseudohernia following herpes zoster infection diagnosed and followed up using ultrasonography. A 60-year-old woman developed right flank pain with vesicular eruptions, treated with valacyclovir. While her pain improved, she noticed new right lateral abdominal bulging. Standing-position ultrasonography revealed relaxation of the right rectus abdominis muscle without fascial defects, confirming an abdominal pseudohernia. Subsequent ultrasounds showed improvement in muscle relaxation, and abdominal bulging visibly reduced.

This case illustrated that point-of-care ultrasonography proved particularly valuable for dynamic evaluation in upright positions and for follow-up of recovery progression, since abdominal pseudohernia manifests as positional muscle relaxation. The modality offers rapid, radiation-free assessment for this rare complication of herpes zoster.

## Introduction

More than 300,000 cases of zoster occur annually in the United States; hence, herpes zoster is a common disease for primary care physicians [1]. After the onset of herpes zoster skin lesions, motor weakness may occasionally develop in the affected segment. This condition, known as segmental zoster paresis, is thought to result from degeneration of motor roots, arising from intense lymphocytic inflammation and vasculitis of the nerves [1]. The association between herpes zoster and motor paresis was first documented by Broadbent et al. in 1866 [2]. Thomas et al. [3] later found motor nerve palsies in 5% of 1,210 patients with herpes zoster. While thoracic involvement was the most common presentation (51.2%, 614/1,200), abdominal motor deficits were rare, occurring in only 0.3% (2/614) of patients with thoracic herpes zoster [3]. Another study reported that the prevalence of segmental zoster paresis among patients with herpes zoster varies between 0% and 6% [4]. Segmental zoster paresis and nerve injury following laparoscopic surgery have been reported to cause thinning of the abdominal wall muscles on ultrasonography or CT [5,6]. The affected muscles become relaxed, allowing intra-abdominal pressure to cause protrusion of the abdominal wall [7]. This condition is termed abdominal pseudohernia and is defined as regional abdominal wall bulging without a fascial defect, in contrast to a true abdominal hernia, which involves protrusion of abdominal contents through a defect in the abdominal wall [7,8]. Muscle paresis also arises from complications such as diabetic neuropathy, ventral root disorders due to disc herniation, and infections such as herpes zoster, Lyme disease, and poliomyelitis [8]. Abdominal pseudohernia is a critical differential diagnosis for patients presenting with postherpetic abdominal bulging, particularly in primary care settings. Because postherpetic abdominal pseudohernia is a rare complication of herpes zoster, it may be overlooked or mistaken for an abdominal hernia, resulting in unnecessary diagnostic investigations and surgical referrals [9]. Diagnosis often relies on clinical history, excluding structural abnormalities by CT imaging [6,10]. However, to our knowledge, only a limited number of reports have described the diagnosis of postherpetic abdominal pseudohernia using ultrasonography alone as the imaging modality [9]. CT and MRI can provide a more detailed evaluation of the suspected area than ultrasonography. However, under conditions associated with increased intra-abdominal pressure, standing-position ultrasonography, which enables a dynamic imaging approach, may be more useful for detecting abdominal wall abnormalities [11]. We present a case of abdominal pseudohernia due to herpes zoster diagnosed by standing-position ultrasonography, which visualized transient rectus abdominis relaxation. 

## Case presentation

**Figure 1:**
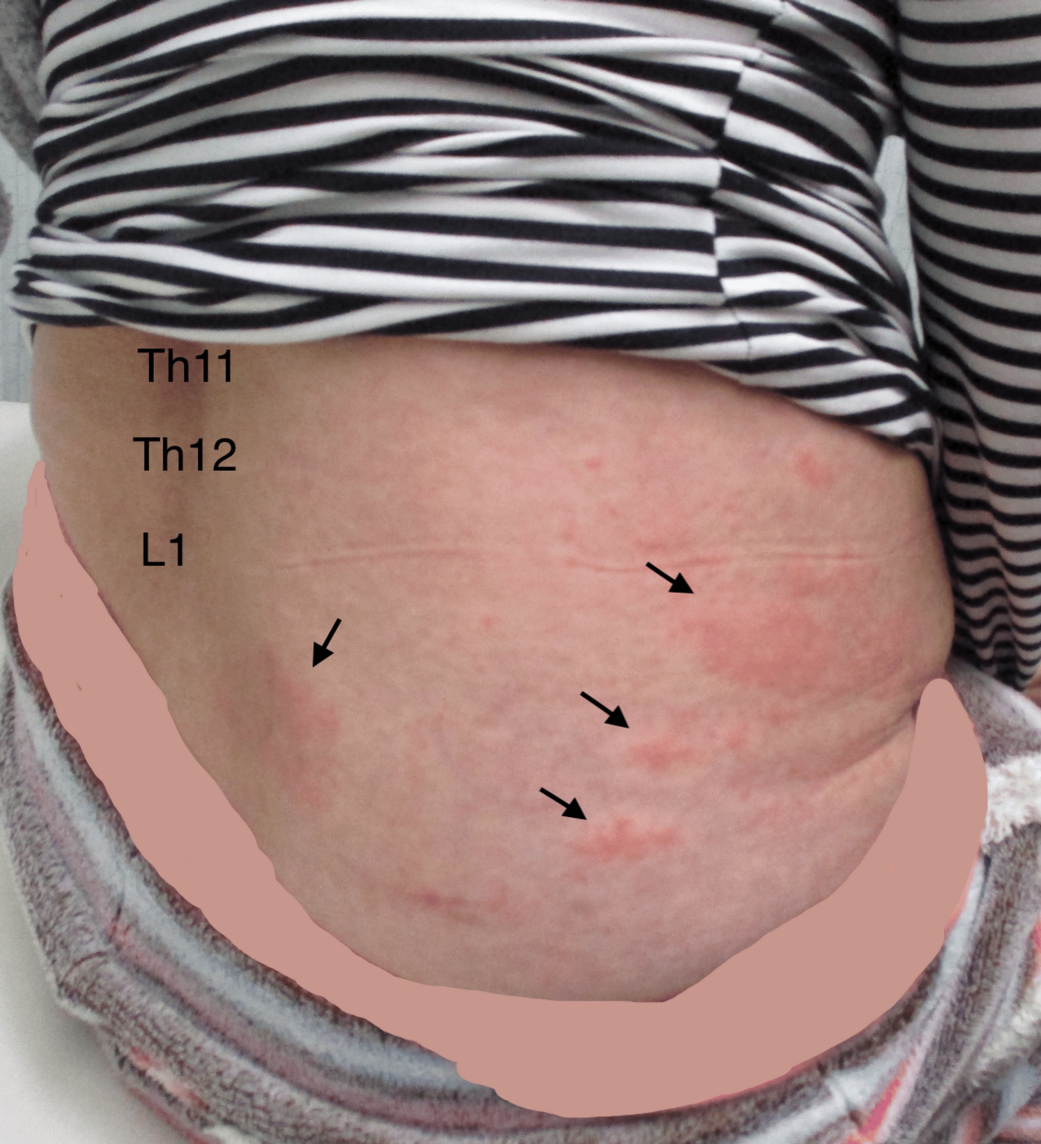
Erythema and vesicles from the lower back to the right side of the abdomen Erythema and vesicles were present from the lower back to the right side of the abdomen at the patient's initial visit. Arrows indicate erythema and vesicles.

A female in her 60s with no significant medical history or regular medications presented with erythema and vesicles extending from the right lumbar region to the right abdomen along the T11-L1 dermatomes (Figure 1).

**Figure 2:**
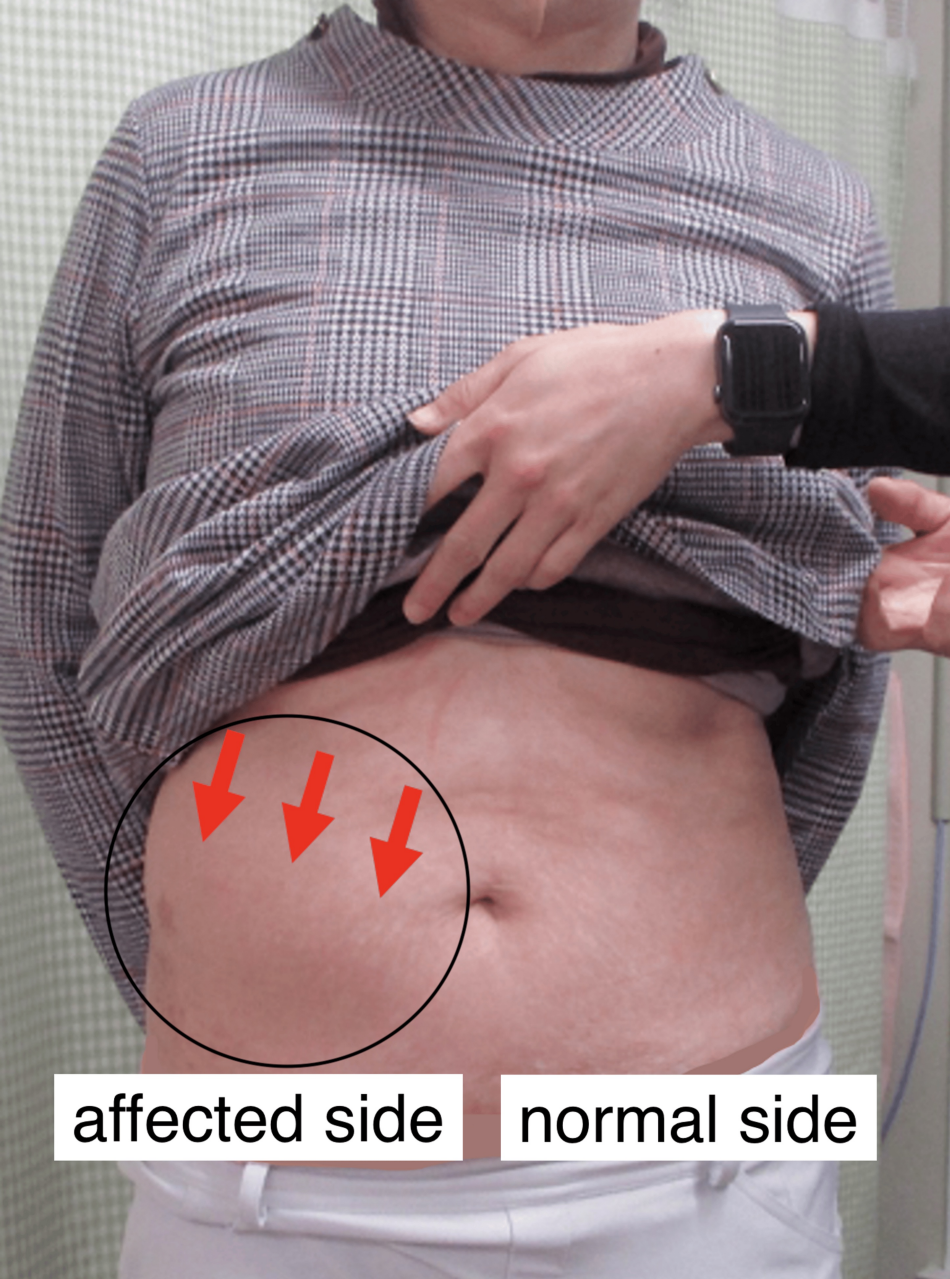
The bulging on the right side of the abdomen The patient’s abdomen presented as bulging on the affected side compared with the contralateral normal side while standing, obtained seven days after the initial visit. Arrows and a circle indicate the bulging.

She was diagnosed with herpes zoster and treated with valacyclovir 1000 mg three times daily for seven days, along with loxoprofen 60 mg three times daily for seven days. Seven days after her first visit, she returned to our hospital because she presented with bulging in the right lower abdomen (Figure 2), and mecobalamin 1500 μg/day was administered for 42 days.

Abdominal physical examination in the supine position found no prominent bulging in the right lower abdomen and no tenderness or any skin appearance indicating a hematoma. Physical examination in the standing position found diffuse bulging in the right lower abdomen but not focal swelling in the limited portion. The overlying skin showed no evidence of skin adherence, such as tethering or induration. She gradually noticed a bulge in the right lower abdomen over several days; it was neither acute nor sudden, nor rapidly progressive. Neurological examination revealed no signs of lower extremity muscle weakness or sensory disturbance, presenting with hypoesthesia in the right lower abdomen. Laboratory data found no particular sign (WBC: 5100 /μL, CRP: 0.34 mg/dL, AST: 34 IU/L, ALT: 21 IU/L, BUN: 15.2 mg/dL, and Cre: 0.66 mg/dL) on day 16 from the first visit. Positional difference about abdominal bulging, the standing position rather than the supine position, enabled us to consider the paresis of the rectus abdominis muscle. Hence, we examined the abdomen using ultrasonography in her standing position. Differential diagnoses for an abdominal bulge include intra-abdominal masses, hematoma in or around the abdominal wall, and true abdominal hernia. As a first step, abdominal imaging is essential to rule out these conditions. Ultrasonographic evaluation demonstrated no evidence of hematoma, protrusion of intra-abdominal organs or masses, or fascial defects of the abdominal wall (Video 1).

**Video 1:** Standing ultrasonographic video of a postherpetic abdominal pseudohernia This video shows the standing patient’s abdomen, obtained seven days after the initial visit, revealing the rectus abdominis evaluated along the body axis on both the affected and unaffected sides. The ultrasonography uses an 11-MHz linear probe. The transverse diameter of the right rectus abdominis was reduced to 2.4-4.7 mm compared with that of the normal left side (approximately 10 mm). (A) The affected side is shown; the arrow indicates the relaxed rectus abdominis. (B) The normal side is shown; the arrow indicates the normal rectus abdominis.

In addition, ultrasonographic video revealed relaxation of the right rectus abdominis muscle compared with the contralateral healthy side (Video 1). This atrophic change served as a useful diagnostic clue for abdominal wall pseudohernia and supported the diagnosis of peripheral motor neuropathy associated with herpes zoster. No surgical intervention was required. Careful subsequent follow-up demonstrated reduced bulging on physical examination, and serial ultrasound examinations showed gradual restoration of rectus abdominis relaxation; however, persistent relaxation of the right rectus abdominis muscle remained evident even after seven weeks of follow-up.

## Discussion

In this case, the precise ultrasound evaluation revealed a reduced transverse diameter of the rectus abdominis muscle affected by paresis due to herpes zoster. Ultrasound diagnosis of motor paresis associated with abdominal wall nerve damage during surgery typically shows that the affected side has a smaller transverse diameter [5]. Previous literatures [3,12] reported that muscle weakness associated with herpes zoster is likely to develop within two weeks of rash onset. On the other hand, abdominal paresis tends to take a longer time, requiring a mean time of 3.5 weeks (from one to eight weeks) [4]. In this case, the pseudohernia appeared 10 days after the onset of abdominal pain, which aligns with the characteristic timeline. Pseudohernia diagnosis generally combines clinical findings with imaging studies. We diagnosed pseudohernia using an imaging device, only ultrasonography. Imaging studies such as CT or MRI are often used to rule out true hernias or organ masses and to identify rectus abdominis atrophy or signal changes in the affected nerve distribution [4,8]. Few studies of pseudohernia have reported the detection of rectus abdominis paresis with a single imaging modality, i.e., ultrasonography.

A notable feature of pseudohernia is that the bulging observed in the standing position may disappear when lying down, suggesting that evaluating patients in both standing and supine positions during imaging could enhance diagnostic accuracy [9,13,14]. An abdominal bulge that is more prominent in the standing position than in the supine position is generally suggestive of an abdominal wall hernia, such as a ventral hernia [15]. The differential diagnosis of ventral hernia includes lipoma, hematoma, seroma, and lymphadenopathy [16,17]. Previous reports have demonstrated that some abdominal wall hernias may be more detectable on ultrasonography when intra-abdominal pressure is increased, such as in the standing position or during the Valsalva maneuver, than conventional imaging such as MRI performed in the supine position [11,18]. In contrast, lipomas, seromas, hematomas, and lymphadenopathy are usually detectable as masses or fluid collections on imaging studies [16]. Ultrasonography is often used in primary care settings, more than CT or MRI. In addition, it is easy to assess patients in various positions, even standing positions. Moreover, the benefits of ultrasound-based diagnosis include rapid assessment and ease of follow-up. We repeatedly followed up on the recovery of pseudohernia using ultrasound, indicating another strength of this modality. These features allow patients to directly observe changes in their condition over time, potentially improving their quality of life. The case with an abdominal bulge after initial work-up to exclude the differential diagnosis of pseudohernia requires follow-up. Pseudohernia presents with relaxation of the rectus abdominis muscle compared with the contralateral healthy side; this relaxation is likely to persist. A literature review [19] reported that approximately 2% of patients with postherpetic abdominal pseudohernia developed serious complications, including visceral herniation and paralytic ileus, which may occasionally necessitate further investigations such as a CT scan or surgical intervention. However, pseudohernia is generally a self-limiting condition, and complete recovery has been reported in 49%-80% of patients within about four months after onset [19]. Given the favorable prognosis and conservative management of most patients with abdominal pseudohernia, dynamic ultrasound incorporating postural changes may be a valuable tool for follow-up. Because this is a single-case report, further studies, including larger case series, are needed to establish the usefulness of standing-position ultrasonography.

## Conclusions

Herpes zoster is a common condition in primary care. Abdominal bulging recovering from herpes zoster should prompt consideration of abdominal pseudohernia as a differential diagnosis. In this case, demonstrating affected muscle relaxation in a standing position by ultrasonography was useful for diagnosing and following up on abdominal pseudohernia.

Ultrasonography offers a unique advantage in evaluating lesions in standing positions, allowing precise identification of the affected area while observing the bulge directly. This makes ultrasonography an effective and practical tool for both diagnosis and follow-up, enhancing patient care and quality of life. Further studies, including larger case series, are required to establish the clinical value of standing-position ultrasonography in the practice of abdominal pseudohernia.
